# The relationship between defecation and feeding in nestling birds: observational and experimental evidence

**DOI:** 10.1186/s12983-015-0116-y

**Published:** 2015-09-11

**Authors:** Rui-chang Quan, Huan Li, Bo Wang, Eben Goodale

**Affiliations:** Center for Integrative Conservation, Xishuangbanna Tropical Botanical Garden (XTBG), Chinese Academy of Sciences (CAS), Menglun, Mengla, Yunnan, 666303 China; College of Forestry, Guangxi University, 100 Daxue Road, Nanning, Guangxi 530005 China

## Abstract

**Background:**

Adult birds clean the nest by consuming or transporting feces, which is thought to be important in order to lower the levels of parasites, pathogens and predation at the nest. If nestlings were to defecate when parents were absent, however, feces could accumulate in the nest.

**Results:**

To understand the mechanism by which nest sanitation is maintained, we studied the timing of defecation in nestling birds of common passerine species in southwest China. In 159 nests of 8 species at the nestling stage during 779 randomly timed observations, we never found fecal sacs present. Video recordings, totaling 455 h at five *Pycnonotus jocosus* nests in the field, showed almost all defecation after feedings, and only nestlings that were fed defecated. Six translocated *P. jocosus* nests were taken into captivity in order to manipulate the frequency of feeding. These nestlings defecated only after feeding, even when feeding intervals were extended to 60 and 120 min. The fecal sac weight also increased with extended feeding intervals, demonstrating a remarkable plasticity for nestlings to wait for feedings.

**Conclusion:**

The evidence allows two major conclusions: 1) defecation in the nest occurs at a time that ensures nest sanitation, stimulated by feeding, rather than there being a set time of gut processing between feeding and excretion; 2) the strong plasticity in the timing of defecation and the possibility of negative repercussions (if defecation occurs when parents are absent) are important mechanisms underlying the efficiency of the feeding-defecation system.

## Introduction

Sanitation behavior is an important strategy for avoiding pathogens and parasites in animals [1] and has been particularly well studied in birds, where a dirty nest could endanger the developing offspring [2]. Parent passerine birds dispose of the fecal sacs of their young by consuming them [3–5], or carrying and dropping them away from their nests [6, 7]. The most often cited advantage of fecal sac disposal is to reduce the exposure of nestlings to parasites and pathogens ([1, 8], reviewed by [2]), as well as to reduce the risk of nestlings being detected by predators through the fecal odor of or any visual cues produced by feces ([9], but see [10]). Although parent birds also could benefit by consuming feces (the “parental-nutrition hypothesis”, [5]), fecal consumption may be costly to adults, through parasite or pathogen transmission [11], and the activity in removing fecal sacs could draw predators’ attention to the nest [12]. Despite these costs, parental removal of fecal sacs is apparently universal among passerine birds, and common in most altricial species [13].

Previous research on fecal sac disposal has focused on the adaptive hypotheses that explain the different kinds of disposal behaviours, but has not quantitatively analyzed the timing and factors eliciting defecation (Mélanie F. Guigueno and William H. Karasov, personal communication), although it has been anecdotally noted that defecation often follows feeding, and in some species parents appear to stimulate defecation by touching the nestlings’ cloaca or beaks [2]. The timing of defecation in relation to disposal is clearly critical, because if defecation occurs over the period when the parent birds are absent and searching for food, the excreta in the nest will soil and contaminate the nest contents. To ensure nests are always in a clean status over the nestling period, we hypothesized that nestlings need to excrete feces at a “right time”, which is the time that would best enable their feces to be disposed of at the moment of excretion. Further, if it can be shown that defecation does take place at the “right time”, how is this regulated, since adults are not always present?

Here we used common passerine bird species of southwestern China to elucidate whether defecation by nestlings occurs during a “right time” and what mechanisms might underlie this phenomenon. We first demonstrate that there were no excreta remaining in nests at any time of the nestling period, suggesting that all feces were immediately disposed of after defecation. We then set out to test how birds achieved this by hypothesizing that defecation is triggered by feeding, rather than occurring after a set period of time since consumption. This hypothesis -- that the timing is regulated by the parents feeding the chicks -- predicts that only the nestling that was fed in a particular feeding bout will defecate. On the other hand, the hypothesis that the timing is regulated by a set processing time in the nestlings’ gut (physiological limit of the nestling birds) predicts that defection will occur no matter whether parent birds are present or absent, and that defecation will occur after a fixed time interval after feeding, which could allow parents to predict when defecation would occur. We investigated these predictions in 5 videotaped nests of the abundant species *Pycnonotus jocosus* in the wild, measuring the time of defecation relative to feedings, and also relative to the last defecation.

Having found that almost all defecations followed feedings, regardless of the interval since the last defecation, we then investigated to what degree the nestlings themselves are able to adjust their defecation to the feeding interval. We brought 6 *P. jocosus* nests from the wild into captivity to manipulate the intervals between feedings and to see how the timing of defecation was affected.

## Results

### Nest survey

In the breeding season of mid-March to August of 2011–2013, we surveyed 134 nests of 6 species, and between April and May 2014, we surveyed 25 more nests of 5 species, for a total of 159 nests of 8 species (Table 1). Each nest contained 1–4 nestlings. No feces were found in nests during the field surveys (*n* = 779 observations of those nests), which were conducted randomly over the nestling period. Nest surveys in 2014 showed that the frequency that adults were present in the nests when they were checked was low (7/157, see Table 1).

**Table 1:** Data from a field survey of feces in nests of passerine birds in southwest China

Species	Number of nests	Visits by observer^a^		Number of observations with feces in nest
		Per nest	Total	
2011-2013				
*Pycnonotus jocosus*	115	1-10	531	0
*P. aurigaster*	12	3-10	59	0
*P. melanicterus*	3	2-7	12	0
*Garrulax chinensis*	2	2-6	8	0
*Copsychus saularis*	1	7	7	0
*Aegithina tiphia*	1	5	5	0
2014				
*P. jocosus*	20	2-10	132 (3)	0
*P. aurigaster*	2	2-8	10	0
*C. malabaricus*	1	2	2 (2)	0
*C. saularis*	1	5	5	0
*Lanius schach*	1	8	8 (2)	0

^a^parentheses indicated number of observations that adults were present in the nest at the time of checking

### Field camera observations

**Fig. 1:**
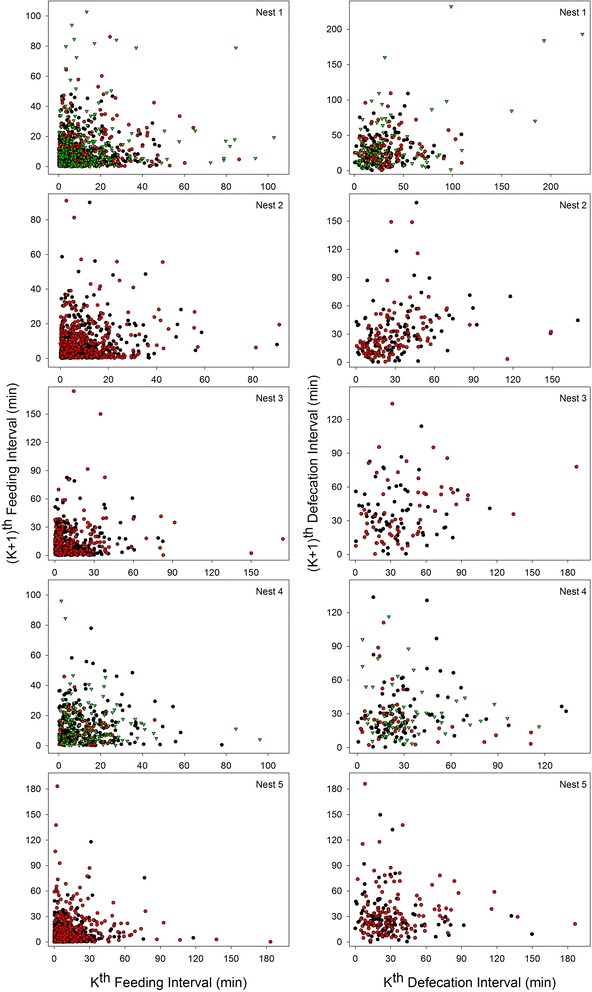
The relationship between the period between the last two events of feeding, or defecation, and the period until the next one, for the five nests observed with video recorders. Colors in each panel indicate different individuals in the same nest. The period between the last two events did not predict the next one (all R^2^ < 0.01)

Five nests were video recorded in the field for a total of 455 h. From these recordings we documented 3787 feeding bouts and 1310 fecal sacs. The intervals between feeding bouts (mean = 11.77 min, SD = 14.25, range = 0.03 - 183.28 min, *n* = 3348) and those between defecations (mean = 32.68 min, SD = 26.80, range = 0.35 - 232.15 min, *n* = 1074) were irregular from one to the next, with the interval from the last feeding or defecation predicting little about the interval to the next one (pseudo R^2^ values for feeding intervals < 0.005; pseudo R^2^ values for defecation intervals = 0.039, Fig. 1).

**Fig. 2:**
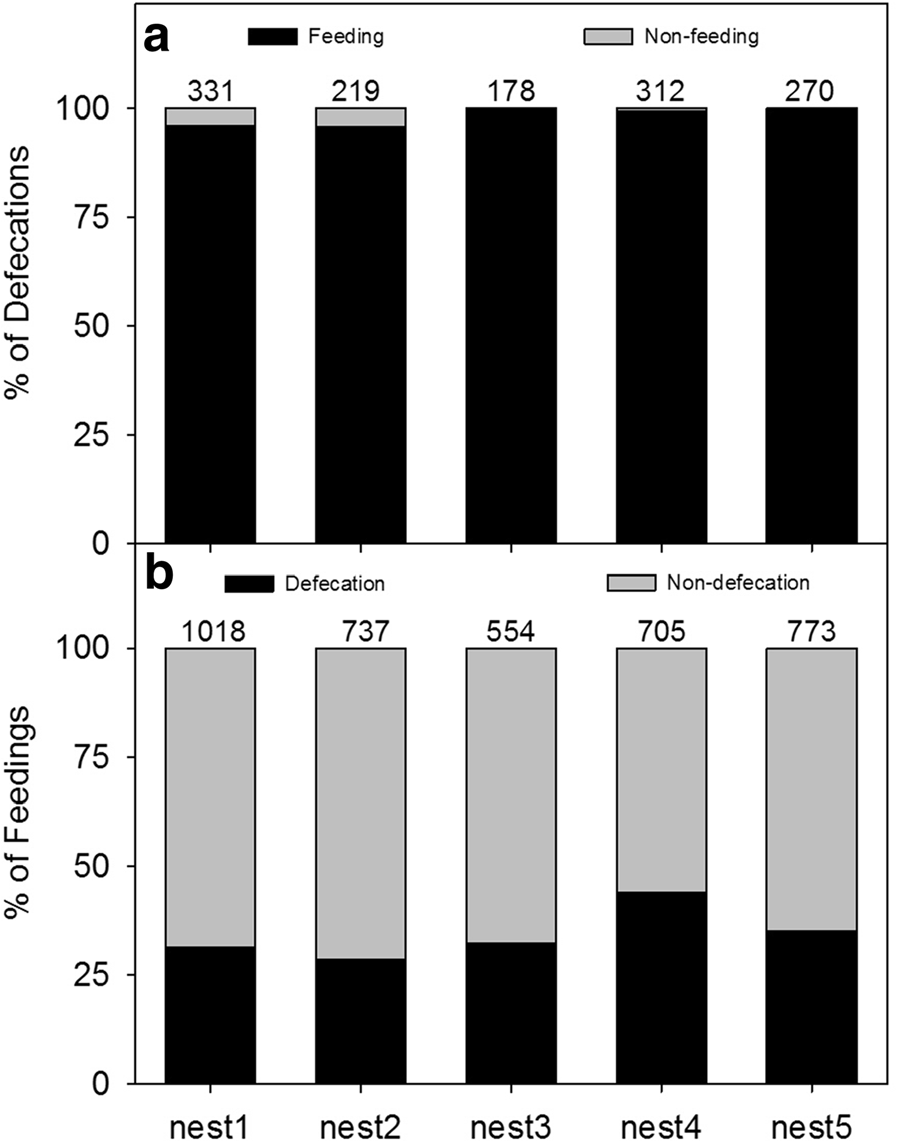
In the field video observations, **a** the proportion of defecations that occurred with feeding (*black portion of bar*; numbers above bars represent the total number of defecations per nest), and **b** the proportion of feedings that occurred with defecation (*black portions of bar*; numbers above bars represent the total number of feedings per nest). Almost all of the defecations occurred with feeding, while only 34.7 % of feedings produced a defecation

Video observation showed that almost all of the fecal sacs (98.2 %, *n* = 1310) were discharged immediately after feeding, but 65.3 % of feedings (*n* = 3787) ended with no defecation (Fig. 2). For the feedings that ended with defecation, the time between feeding and defecating was about 6.4 s (SD = 2.8, *n* = 300). Adults caught the sacs directly from the nestling’ cloacae, and thus there was no time interval between defecation and disposal, if defecation occurred during feeding. For the feedings that ended without defecation, adult birds spent an average of 12.9 s (SD = 7.7, *n* = 300) waiting for a defecation before leaving the nest. Totally 24 sacs (1.8 %, *n* = 1310) occurred when the parents were absent, which were directly dropped into the nest, with the parents disposing of them a mean of 12.6 min later (SD = 12.9, *n* = 16; the amount of time for parent birds to dispose of another 8 sacs were not documented by film because the disposal behavior happened over the non-observation period of night, 7 PM and 8 AM).

For the 1286 sacs that occurred during feeding, the videos showed that usually one nestling was fed for each feeding bout, and that only the nestlings that were fed defecated. Other nestlings of the same nest that were not fed never excreted feces during the same feeding bout (3787 feeding observations). This demonstrates that the parents’ presence near the nestlings is not enough to stimulate defecation.

### Laboratory experiments

**Fig. 3:**
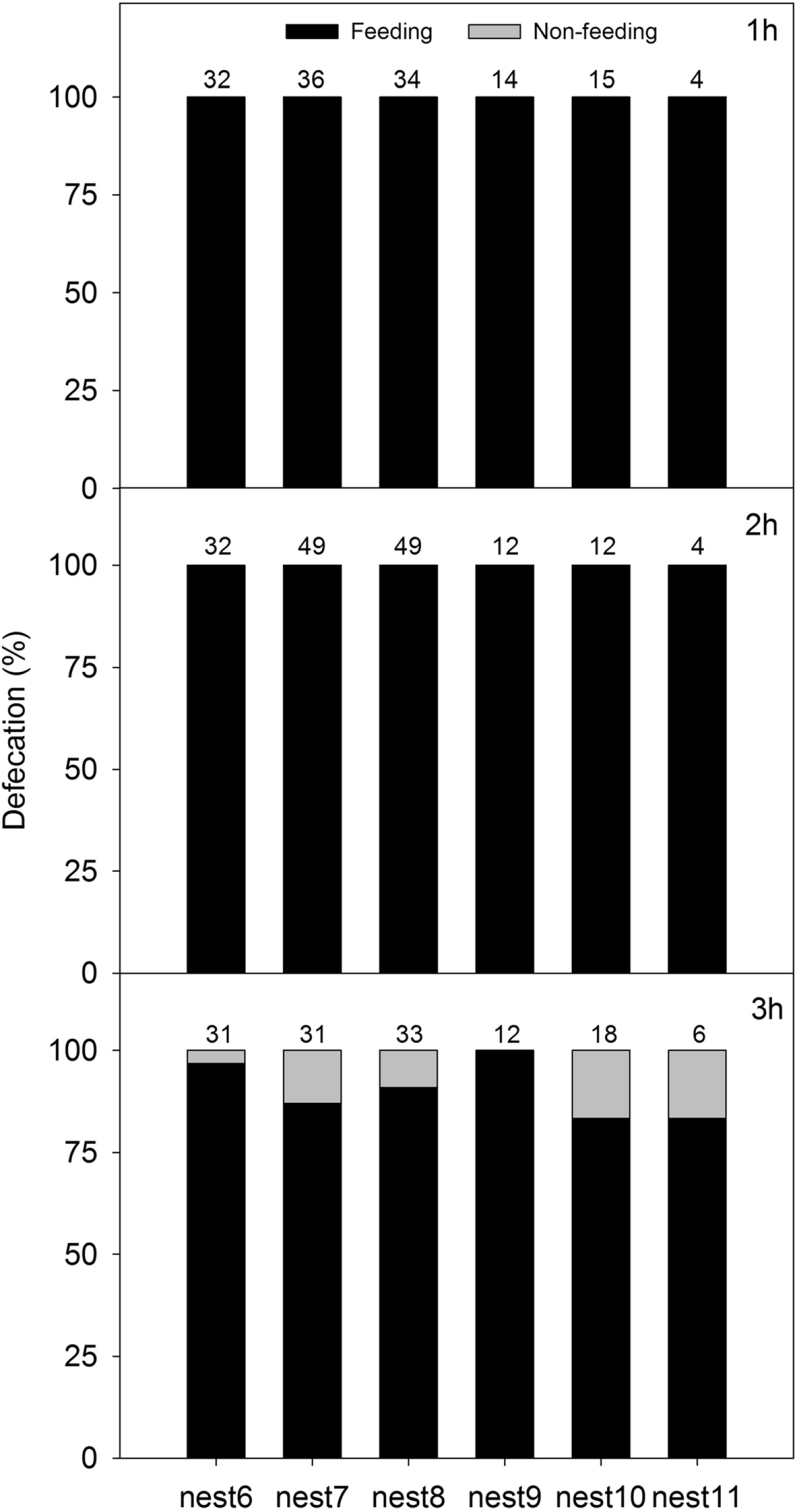
In the laboratory experiments, the percentage of fecal sacs defecated during feeding (*black portion of bar*). Nests 6–8 each contained 2-day old nestlings; nests 9–11 each contained 6-day old nestlings. Numbers above bars represent the total number of defecations by that nest in that treatment

**Fig. 4:**
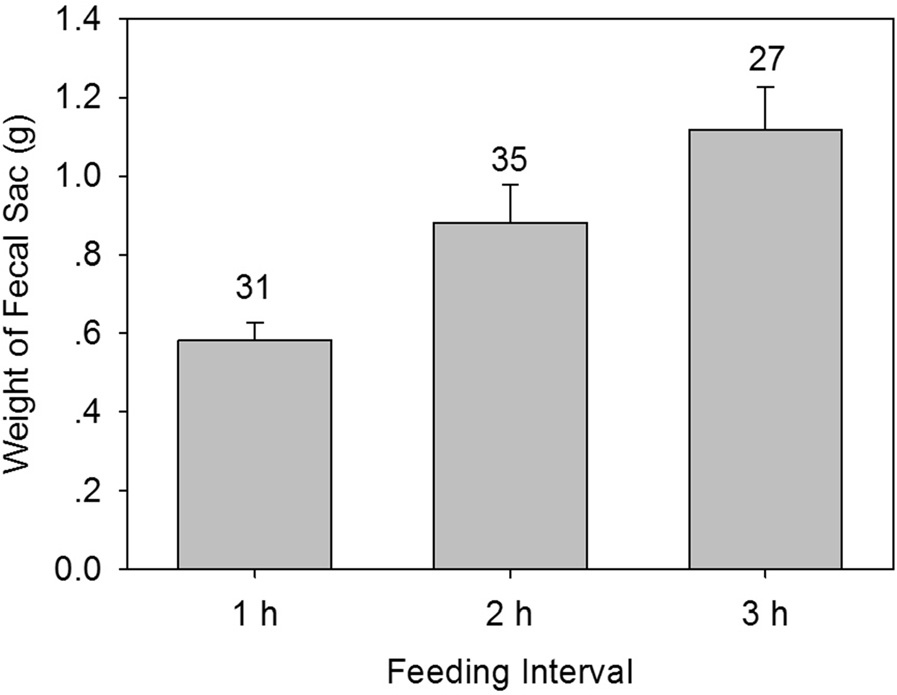
The average weight of fecal sacs (± SE) increased with the time interval of feeding in the laboratory experiments. The numbers above bars indicate the number of data points. Each data point is a nestling that defecated during the particular treatment

In the laboratory experiments, two-day old nestlings and six-day old nestlings had a similar percentage of defecation during feeding (Fig. 3). When fed with time intervals of 1 h and 2 h, nestlings excreted all of the fecal sacs during the 1–2 min feeding period. When nestlings were fed with 3 h time interval, 91 % (*n* = 133 observations from the 6 nests) of fecal sacs were excreted during the feeding period (see Fig. 3). Interestingly, the wet weight of fecal sacs increased with longer feeding intervals (*X*^*2*^_2_ = 18.19, P = 0.0002, Fig. 4).

## Discussion

This study was not designed, as most previous research has been, to assess the adaptive benefits of nest sanitation. Rather, we asked the question of what is the right time of defecation, and further, what is the mechanism that guarantees all of the defecation to occur at the right time.

The field nest survey (8 bird species, 159 nests, and 779 observations) showed that no excreta remained in nests at any time of the nestling period. This suggests that feces were excreted at the hypothesized “right time”, the time following feeding that enable feces to be disposed immediately. Is it possible that the timing of defecation was random with respect to feeding, and the clean nests are the result of parents removing feces quickly? Recent research has demonstrated that adults put a lot of energy into removing feces, and sometimes when there are too many fecal sacs to remove in one flight away from the nest, birds will make repeated visits to the nest to remove feces, without bringing more food [14]. However, parents are often away from the nest: in the last year’s data we noted that adults were relatively infrequently present at the nest (during 7 of 157 visits, although we should acknowledge there is a chance that adults might have left the nest when hearing our approach, before we detected them). Hence, if defecation occurred randomly with respect to feeding and then feces were removed by the parents, we would still have expected to find some feces in the nest in this sample of observations.

From the data we collected at the five videotaped nests, the timing of the next defecation was not predictable based on the period between the last two defecations. It is possible that parents have greater knowledge about the situation (e.g., how big and easily digestible the meal was that they delivered) and could hence predict when their young would be ready to defecate again. Yet it is most parsimonious to hypothesize that defecation is simply regulated by feeding: in the video recordings, the nestlings usually defecated shortly (mean 6.4 s) after a feeding. Such a short feeding-defecation interval indicates that the defecation is not the digested remains of the food that was just fed; rather it is stored fecal material from earlier feedings. For example, in other experiments, we have shown that Red-whiskered Bulbuls excrete seeds between 15 and 40 min (on average, for six species of fruits) after consuming them [15].

As nestlings always complied with the rule that if there was no feeding, there was no defecation, it strongly suggests that feeding is the stimulus that releases defecation. Adults in this species collected the feces directly from cloacae, leaving no possibility of nest pollution. However, in the field, the feeding decision of parents are complex and context dependent as to which of its nestlings to feed ([16], and reference therein), and if a nest has more than one nestling, this means that other siblings of the same nest that were not fed have to retain their sacs until they are fed. Are nestlings able to adjust the physiological process of defecation to wait until they are fed?

Our laboratory experiment showed that nestlings’ excreting time is much more plastic than we expected: nestlings can wait for at least 2 h to defecate (Fig. 3), and this is a much greater interval of defecation – more than ten times – than what occurred in the field. In addition, nestlings produced heavier fecal sacs associated with the longer feeding intervals. The combined results thus show coordination between the two partners, the parents and nestlings in maintaining the feeding-defecation system: the parents’ feeding is necessary (as shown by the fact that only the nestlings that were fed defecated), but the nestlings also have the ability to modulate their own physiological processes to wait for the parents to feed. This large plasticity in defecation time could allow parents to feed nestlings infrequently in times of stress, such as when a predator is present (e.g., [17]).

Finally, our observations also show that if excrement occurs at the wrong time – when parents are absent – both nestlings and parents could bear possible negative consequences. For example, we observed some parent birds in our study spent extra time searching for the excrements that occurred in their absence after they returned to the nest, thus most likely decreasing their foraging time and resting time. It seems the possible negative consequences (less feeding for nestlings; less rest for adults; threats of pathogens, parasites and predators) if defecation occurs during adult absent may also be an important factor maintaining the effectiveness of the feeding-defecation interaction between adults and nestlings.

## Conclusions

Our observations showed that almost all of the defecation by nestling birds occurred at a “right time”, immediately after feeding. The feeding-defecation system is efficient in avoiding nest pollution, because parents dispose of all the sacs directly when they feed the nestlings. Further, we provide the first quantitative evidence of parent-nestling coordination in this system: parents provide the feeding, but at the same time the nestlings are able to modify their physiological process to wait for the parents to come and feed. The strong plasticity in defecation time and the possibility of negative repercussions if defecation occurs when adults are absent are important forces driving defecation to occur with feeding, even if the feeding rate changes greatly during one day.

## Materials and methods

### (a) Nests survey and bird observation in the field

A field survey of nesting birds was conducted over the breeding season from mid-March to late August, 2011–2013, at the Xishuangbanna Tropical Botanical Garden (XTBG; centred at 21°55′N, 101°16′E) in southwest China. Nests were identified to species by direct observation after they were found. Each nest was checked randomly 1–10 times over the breeding period to record whether nestling feces was present. As each check was conducted quietly and quickly (often less than 10 s), and one time a day to decrease disturbance; no nests were abandoned because of our visitation. A similar survey was conducted in XTBG between April-May 2014, in which we recorded not only feces, but also whether adult birds were present in the nest at the time it was checked.

To identify the potential factors that regulated the defecation time under natural conditions, we conducted detailed observations on five nests of the Red-whiskered Bulbul (*Pycnonotus jocosus*) in the field (mean = 2.4 nestlings/nest, SD = 0.55, *n* = 5), because this species is abundant and its nests are accessible for observation, often being less than 2 m above the ground. The field observation was conducted using a digital video camera (Sony HDR-XR550), placed at a distance of 0.5-2 m to the nests. Nestlings were marked by the use of non-toxic paints on the top of their heads for individual identification. The first 20 min of observation was excluded from the analysis to permit the parents to return to their normal behaviour. Films were later viewed to document the time of each feeding and defecation, and the status of parent birds (present or absent) when defecation occurred. If only one nestling was fed at a feeding bout, we investigated whether only it defecated. All videotaped individuals fledged.

### (b) Laboratory experiment

To investigate the degree of control nestlings have in waiting to defecate after a feeding, we turned to a more controlled laboratory environment, in which the “parents” were people feeding the birds.

We relocated 6 different *P. jocosus* nests to the laboratory (mean = 2.7 nestlings/nest, SD = 0.52, *n* = 6), three nests each containing 2 day old nestlings and three nests containing 6 day old nestlings. Each nest was fed for between 1 and 2 min at 1 h, 2 h and 3 h time intervals consecutively in that order, and then this feeding protocol was repeated. Nestlings were fed the same food quantity and items at each feeding; food consisted of primarily mealworms and fruit pellets, comparable to the diet fed to nestlings by parents. We watched the young birds for evidence of undernourishment under these conditions, but their growth appeared similar to the wild offspring of the same age and all these laboratory raised birds were successfully fledged. They were then kept in an aviary for separate experiments.

The data we collected in this experiment included the number of sacs excreted during feeding and non-feeding periods. For the nests containing 2 day old nestlings the experiment lasted 6 days, and for the nests with 6 day old nestlings the experiment lasted 2–3 days. After each feeding visit, we removed all the fecal sacs in the nest to keep it clean. For the 2 day old nestlings, we determined the wet weight of each fecal sac (nearest 0.01 g) at about 10–20 min after they were collected. To increase the sample size for this fecal sac weight data, we moved one additional Red-whiskered Bulbul nest to the lab when the nestlings were two days old (hence the fecal weight data has sample size *n* = 4 nests).

This research was approved by the Administrative Panel on the Ethics of Animal Experiments of the Xishuangbanna Tropical Botanical Garden, Chinese Academy of Sciences (protocol: XTBG 2011–003), and strictly adhered to the Guideline for the Care and Use of Laboratory Animals in China.

### (c) Statistical analysis

The time interval of feedings was calculated as the amount of time between two consecutive feedings of the same individual; the time interval of defecation was also the amount of time between two consecutive defecations for the same nestling. We then asked if there was a relationship between a feeding or defecation interval and the one that followed it. We used a general linear mixed model, with the earlier interval as the fixed factor and nestling (nested within nest) as a random factor. To calculate a pseudo R-squared for these models, we followed Nakagawa and Schielzeth [18].

To analyze whether in the laboratory experiment treatment affected fecal wet weight, we also applied a general linear mixed model. For this model, treatment was the fixed factor, and nest was the random factor (data was not available per individual nestling).

Generalized linear mixed models were implemented using the lme4 package [19] in R (R Core Team, version 3.1.2, 2014).
